# Inhibitory Effects of Resveratrol on PDGF-BB-Induced Retinal Pigment Epithelial Cell Migration via PDGFRβ, PI3K/Akt and MAPK pathways

**DOI:** 10.1371/journal.pone.0056819

**Published:** 2013-02-14

**Authors:** Chi-Ming Chan, Hsun-Hsien Chang, Vin-Chi Wang, Chuen-Lin Huang, Chi-Feng Hung

**Affiliations:** 1 Department of Ophthalmology, Cardinal Tien Hospital, New Taipei City, Taiwan; 2 School of Medicine, Fu-Jen Catholic University, New Taipei City, Taiwan; 3 Children's Hospital Informatics Program, Harvard-Massachusetts Institute of Technology Division of Health Sciences and Technology, Harvard Medical School, Boston, Massachusetts, United States of America; 4 Neurological Center, Cardinal Tien Hospital, New Taipei City, Taiwan; 5 Medical Research Center, Cardinal Tien Hospital, New Taipei City, Taiwan; 6 Department of Physiology and Biophysics, Graduate Institute of Physiology, National Defense Medical Center, Taipei City, Taiwan; Chang Gung University, Taiwan

## Abstract

**Purpose:**

In diseases such as proliferative vitreoretinopathy (PVR), proliferative diabetic retinopathy, and age-related macular degeneration, retinal pigment epithelial (RPE) cells proliferate and migrate. Moreover, platelet-derived growth factor (PDGF) has been shown to enhance proliferation and migration of RPE cells in PVR. Even resveratrol can suppress the migration and adhesion of many cell types, its effects on RPE cell migration and adhesion remain unknown. In this study, we investigated the inhibitory effects of resveratrol on RPE cell migration induced by PDGF-BB, an isoform of PDGF, and adhesion to fibronectin, a major ECM component of PVR tissue.

**Methods:**

The migration of RPE cells was assessed by an electric cell-substrate impedance sensing migration assay and a Transwell migration assay. A cell viability assay was used to determine the viability of resveratrol treated-cells. The cell adhesion to fibronectin was examined by an adhesion assay. The interactions of resveratrol with PDGF-BB were analyzed by a dot binding assay. The PDGF-BB-induced signaling pathways were determined by western blotting and scratch wound healing assay.

**Results:**

Resveratrol inhibited PDGF-BB-induced RPE cell migration in a dose-dependent manner, but showed no effects on ARPE19 cell adhesion to fibronectin. The cell viability assay showed no cytotoxicity of resveratrol on RPE cells and the dot binding assay revealed no direct interactions of resveratrol with PDGF-BB. Inhibitory effects of resveratrol on PDGF-BB-induced platelet-derived growth factor receptor β (PDGFRβ) and tyrosine phosphorylation and the underlying pathways of PI3K/Akt, ERK and p38 activation were found; however, resveratrol and PDGF-BB showed no effects on PDGFRα and JNK activation. Scratch wound healing assay demonstrated resveratrol and the specific inhibitors of PDGFR, PI3K, MEK or p38 suppressed PDGF-BB-induced cell migration.

**Conclusions:**

These results indicate that resveratrol is an effective inhibitor of PDGF-BB-induced RPE cell migration via PDGFRβ, PI3K/Akt and MAPK pathways, but has no effects on the RPE cell adhesion to fibronectin.

## Introduction

Cells of the retinal pigment epithelium (RPE) form a highly specialized monolayer between Bruchs membrane and the choroid on their basal side and the neurosensory retina on their apical side. The cells play important roles in eye development and visual function. They allow transportation of nutrients from the choroid to the photoreceptors, recognition of the light cycle, maintenance of the blood–retinal barrier, and phagocytosis of shed photoreceptor outer segments. In diseases such as age-related macular degeneration (AMD)[1], proliferative vitreoretinopathy (PVR)[2], [3], [4] and proliferative diabetic retinopathy (PDR)[5], RPE cell migration may result in severe visual impairment[5]. RPE cell migration is a complex molecular process regulated by growth factors and cytokines. Among the growth factors, PDGF exhibits more chemotactic and proliferative effects than others on RPE cells[6], is important for development of PVR and fibrovascular membrane (FVM) in PDR[7].

Resveratrol (3,5,4′-trihydroxystilbene), a major polyphenol found in grapes, red wine, peanuts, and other plants[8], [9], has been shown to be involved in antioxidant[10], [11], anti-proliferative[12], [13], [14], anti-inflammatory[15], [16] and chemopreventive[17] activities. A number of potential health benefits, including reduced risk of cancer[18], [19], [20] and heart disease[21], [22], are thought to be associated with consumption of resveratrol. However, its effects on the retina have not been well documented.

Our previous studies have demonstrated that lycopene and epigallocatechin gallate (EGCG) can inhibit PDGF-BB-induced signaling and migration of adult human retinal epithelial (ARPE19) cells through direct binding with PDGF-BB[23], [24]. In this study, we investigated the inhibitory effect of resveratrol on PDGF-BB-induced ARPE19 cell migration and the potential mechanisms involved. These mechanisms include the influence of resveratrol on ARPE19 cell adhesion, viability, the expression of anti-phosphotyrosine antibodies (4G10), platelet-derived growth factor receptor β (PDGFRβ), phosphatidylinositol-3 kinase (PI3K)/Akt pathway activation and mitogen-activated protein kinase (MAPK) activation.

## Materials and Methods

### Materials

Resveratrol, bovine serum albumin (BSA), aprotinin, leupeptin, phenylmethylsulfonyl fluoride (PMSF), sodium fluoride (NaF), sodium orthovanadate, mitomycin-C, LY294002, U0126, SP600125 and SB203580 were purchased from Sigma Chemical Co. (St Louis, MO, USA). AG1295 was purchased from Merck KGaA. (Darmstadt, Germany). Human plasma fibronectin was from Life Technologies Corporation (Carlsbad, CA, USA). Antibodies (Ab) raised against PDGFRβand phospho-extracellular signal-regulated kinase 1/2 (ERK1/2) were from Santa Cruz Biotechnology (Santa Cruz, CA, USA). Ab raised against phosphotyrosine (4G10) was from EMD Millipore Corporation (Billerica, MA, USA). Abs raised against phospho-PDGFRα, phospho-PDGFRβ, phospho-PI3K, phospho-Akt, Akt, phospho-c-Jun N-terminal kinase (JNK), phospho-p38 and p38 were from Cell Signaling Technology, Inc. (Beverly, MA, USA). Abs raised against total ERK1/2 and JNK were from R&D systems, Inc. (Minneapolis, MN, USA).

### Cell cultures

ARPE19 cells were purchased from Food Industry Research and Development Institute (Hsinchu, Taiwan) and were maintained in DMEM/F12 supplemented with 10% fetal bovine serum (GibcoBRL, Invitrogen Life Technologies, Carlsbad, CA), 100 units/ml penicillin, and 100 mg/ml streptomycin (Sigma Chemical Co., St. Louis, MO). The cells were cultured in a humidified incubator at 37°C and 5% CO_2_. For most of the experiments, cells reaching a 90%–95% of confluence were starved and synchronized in serum-free DMEM/F12 for 24 hours before they were subjected to further analysis.

### Resveratrol treatment and PDGF-BB incorporation

Resveratrol was dissolved in dimethyl sulfoxide (DMSO) to the desired concentrations. In migration assays and the Western blot analysis, the serum-free cell culture medium with various concentrations of resveratrol were all preincubated with or without PDGF-BB (20 ng/ml) at 37°C for 30 minutes.

### ECIS migration assays

*ECIS migration assays* were conducted with the Electric Cell-Substrate Impedance Sensing (ECIS) system (Applied Biophysics, Inc., Troy, NY). ARPE19 cells were cultured in 8W1E ECIS arrays (Applied Biophysics), where each well for cell culture contained a small gold film circular electrode (5×10^−4^ cm^2^) and a larger (0.15 cm^2^) counter electrode. The electrodes were connected to the ECIS 1600R instrument that applied an approximately constant AC current (1 µA at 4,000 Hz) between the two electrodes using culture medium as the electrolyte. The instrument monitored both the voltage across the electrodes and the phase relative to the applied current. From this information, the instrument reported the impedance, resistance and capacitance of the small electrode, treating the system as a series RC circuit. As the cells attached and spread on the small electrode, their membranes constricted the current and forced it to flow in the space beneath the basal membrane and the electrode surface and in the paracellular path between adjacent cells (the barrier function), resulting in a significant increase in impedance. The microampere current and the resulting voltage drop of a few millivolts have been shown to have no measurable effect on the cells, and hence, the monitoring of cell behavior was noninvasive [25].

We seeded ARPE19 cells at a concentration of 70,000 cells/well in the arrays and incubated them for 24 hours. The experiments were conducted on wells where the electrode resistance had achieved a steady state. After being submitted to an elevated voltage pulse of 40-kHz frequency, 4-V amplitude, and 10-s duration, which led to death and detachment of cells present on the small active electrode, the medium was changed to a serum-free cell culture medium containing various concentrations of resveratrol preincubated with or without PDGF-BB (20 ng/ml) at 37°C for 30 minutes. Cells surrounding the small active electrode that had not been submitted to the elevated voltage pulse then migrated inward to replace the killed cells. Cell migration was assessed by continuous resistance measurements for 30 hours.

### Transwell migration assays

Transwell migration assays with ARPE19 cells were performed by using a modified Boyden chamber model (Transwell apparatus, 8.0 mmpore size, Costar)[26]. For detection of ARPE19 cell migration in the Transwell, the lower face of polycarbonate filters (Transwell insert) were coated with fibronectin (0.3 mg) for 30 minutes in the laminar flow hood. The lower chamber was filled with 0.6 ml of serum-free medium or PDGF-BB (20 ng/ml)-containing medium which was preincubated with various concentrations of resveratrol. ARPE19 cells (5×10^4^ cells, 200 µl) were plated to the upper chamber. After 5 hours of incubation, all cells that had not migrated were removed from the upper face of the Transwell membrane with a cotton swab and migrated cells were fixed and stained with 0.5% toluidene blue in 4% PAF. Migration was quantified by counting the number of stained cells per ×100 field (high power field, HPF) with a phase-contrast microscope (Leica DMIL1) and photographed.

### Cell viability assays

The viability of cells was determined by the MTT. The MTT assay in our laboratory has been previously described[27]. Briefly, resveratrol-treated cells were incubated for 24 hours. After a brief wash with medium, 0.5 mg/ml MTT in DMEM was used for the quantification of living and metabolically active cells. Mitochondrial dehydrogenases metabolized MTT to a purple formazan dye, which was analyzed photometrically at 550 nm. Cell viability was proportional to the absorbance measured.

### Adhesion assays

96-well plates were coated with 50 µl fibronectin (15 µg/ml in PBS, pH 7.4) per well and incubated at 37°C for 24 hours. After being washed with PBS three times, the unspecific binding of fibronectin on the plates was blocked by 100 mg/ml bovine serum albumin (Sigma-Aldrich) in PBS at room temperature for 1 hour. ARPE19 cells were trypsinized and resuspended in serum-free cell culture medium and labeled with BCECF/AM (10 mg/ml) for 30 minutes at 37°C. After being washed with the serum-free cell culture medium, the labeled cells were resuspended in the serum-free cell culture medium with different concentrations of resveratrol to a density of 1.0×10^5^ cells/ml and incubated for another 30 minutes at 37°C. Then the suspended cells were applied onto 96-well plates within 100 µl serum-free cell culture medium with different concentrations of resveratrol per well and incubated at 37°C for 1 hour. After being gently washed with PBS three times, the nonadherent cells were removed by aspiration and the 96-well plates were subjected to measurement by Wallac Victor 3 1420 multilabel counter (Perkin Elmer, Turku, Finland) using excitation and emission wavelength of 485 and 535 nm, respectively.

### Dot binding assay

A nitrocellulose membrane (Bio-Rad Laboratories, Hercules, CA) was soaked in a buffer (25 mM Tris, 192 mM glycine and 20% methanol) for 30 seconds. Recombinant PDGF-BB (2 µg/ml in 50 µl) was applied to the membrane with a Bio-Dot microfiltration apparatus (Bio-Rad Laboratories, Hercules, CA) by suction. 2.5 µl of DMSO, 3 µM and 10 µM of resveratrol were directly spotted on the same membrane. The membrane was then blocked with BSA (5% in PBS) for 0.5 hour. After being washed with PBS, the membrane was incubated with PDGF-BB (0.5 µg/ml) in PBS for 1 hour at room temperature (RT). A brief wash was followed, and the membrane was then incubated with anti-PDGF-BB Ab (2 µg/ml in 1% BSA-containing PBS) for 1 hour at RT. After another brief wash, the membrane was incubated with horseradish peroxidase-conjugated Ab before being developed by enhanced chemiluminescence (ECL; NEN, Boston, MA).

### Cell lysate preparation and Western blot analysis

ARPE19 cells cultured on 6 cm dishes were starved for 24 hours and then treated with various concentrations of resveratrol which were preincubated with or without PDGF-BB (20 ng/ml) at 37°C for 30 minutes. After 10 or 30 minutes of further incubation, the ARPE19 cells were washed with PBS twice and the phosphorylation of the tyrosine (4G10), PDGFRα, PDGFRβ, PI3K, Akt, ERK, JNK and p38 were analysed. They were then lysed in radioimmunoprecipitation assay buffer [17 mM Tris–HCl, pH 7.4, 50 mM NaCl, 5 mM EDTA. 1 mM sodium fluoride, 1% Triton X-100, 1% sodium deoxycholate, 0.1% SDS, 1 mM sodium orthovanadate, 1 mM PMSF, and 1 µg/ml aprotinin and leupeptin (freshly prepared)]. After sonication, the lysate was centrifuged (14,000 *g* for 10 minutes at 4°C), and the supernatant was removed. The protein content was quantified by a Pierce protein assay kit (Pierce, Rockford, IL). Total protein was separated by electrophoresis on 8% SDS–polyacrylamide gels. The proteins were then electroblotted onto polyvinylidene fluoride PVDF membranes and probed using the indicated antibodies. Immunoblots were detected by enhanced chemiluminescence (Chemiluminescence Reagent Plus from NEN, Boston, MA). For some of the experiments, the PVDF membrane was stripped at 60°C for 30 minutes with a stripping buffer (62.5 mM Tris-HCl, pH 6.7, 2% SDS and 100 mM β-mercaptoethanol).

### Scratch wound healing assay

In vitro scratch wound healing assay was created by scraping the cell monolayer with a linear scratching with a sterile 20- µl pipette tip. The cells were immediately washed and wound closure with various treatments was monitored for a maximum of 16 h followed by photography under phase-contrast microscope (Leica DMIL1). Assays were performed in the presence of mitomycin-C (5 µg/ml) for 1 hour to prevent proliferation of RPE cells. Migration was quantified by counting the number of migrated cells per ×100 field (high power field, HPF).

### Statistical analysis

All data were analyzed with SigmaPlot for Windows (Version 10.00). Data are expressed as mean ± standard error (SE) of four experiments. Comparison of means between two groups of data was made using the unpaired, two-tailed Student *t* test.

## Results

### Resveratrol inhibited PDGF-BB-induced ARPE19 cell migration

**Figure 1:**
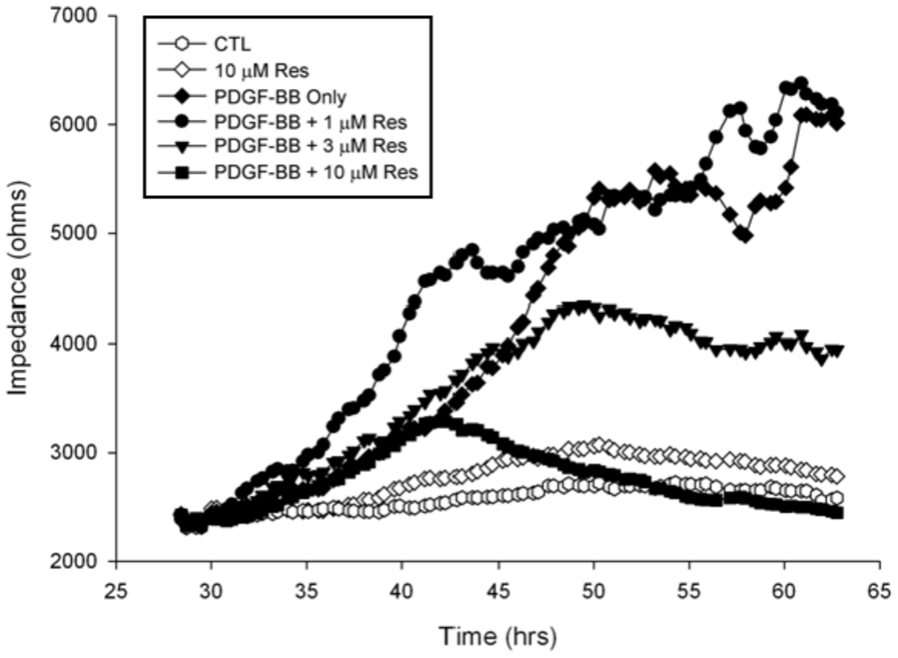
PDGF-BB-induced ARPE19 cell migration was inhibited by resveratrol in an ECIS migration assay. The cells cultured in 8W1E ECIS arrays were treated with different combinations of PDGF-BB (20 ng/ml) and resveratrol (CTL indicates that it contained only DMSO) which were preincubated together at 37°C for 30 minutes. Cell migration was then assessed by continuous resistance measurements for 30 hours. Resveratrol (Res) (10 µM) did not increased cell migration when PDGF-BB was not present. In the well containing PDGF-BB but not resveratrol, the impedance, which corresponds to the number of cells migrated to the surface of the detective electrode, increased sharply during the first 10 hours. By contrast, the impedance in the well containing PDGF-BB and resveratrol increased slowly during the same time period.

**Figure 2:**
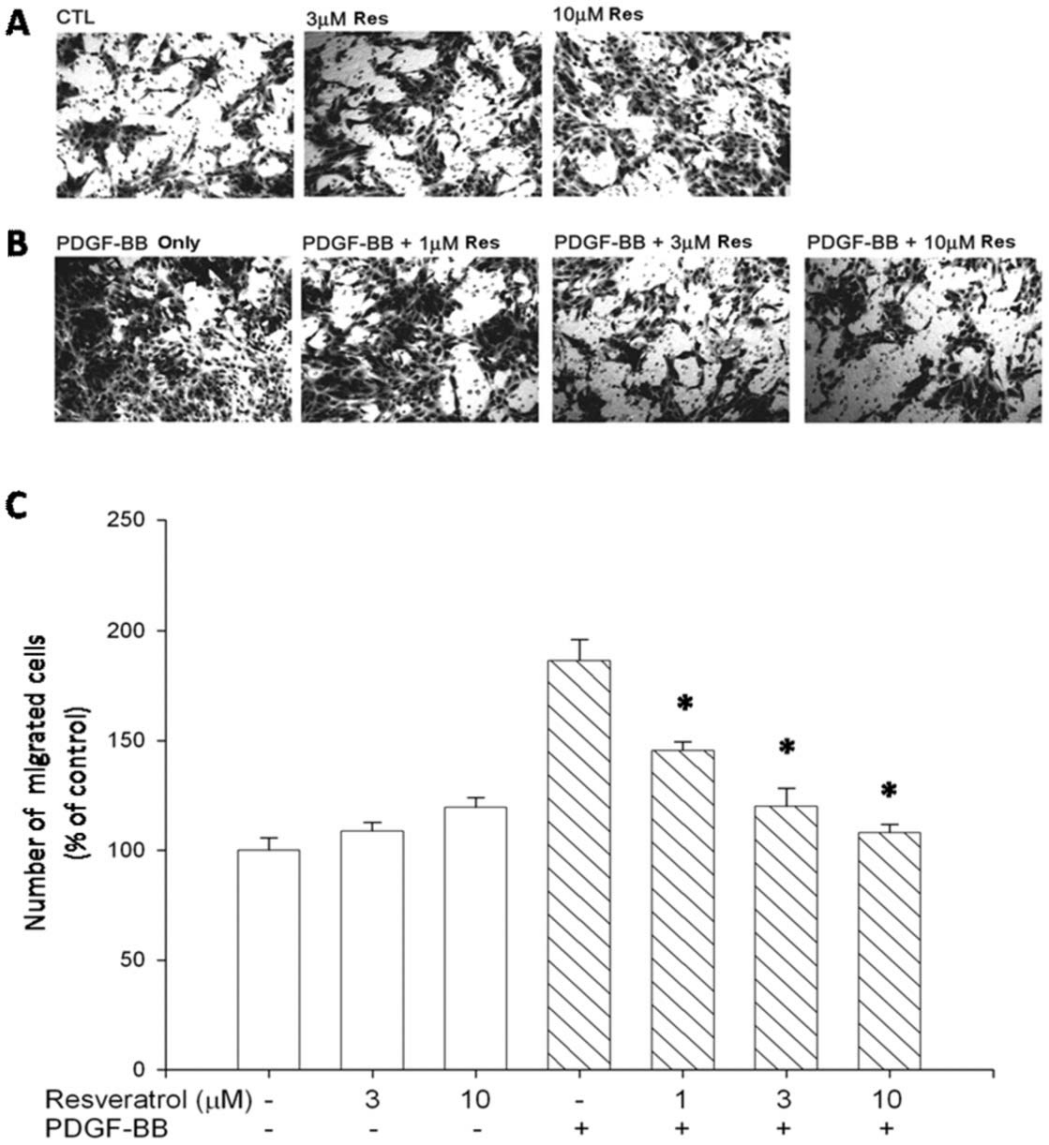
Transwell migration assay showed that PDGF-BB-induced ARPE19 cell migration was inhibited by resveratrol. Transwell inserts were coated with fibronectin (0.3 mg). ARPE19 cells (5×10^4^ in 200 µl) were seeded in the upper chamber in the absence or presence of resveratrol. The inserts were assembled in the lower chamber, which was filled with 600 µl serum-free medium without PDGF-BB (A) and containing PDGF-BB (20 ng/ml) (B), and preincubated with various concentrations of resveratrol for 30 mininutes at 37°C. After incubating for 5 hours at 37°C, fixation was performed. ARPE19 cells that migrated to the underside of filter membrane were photographed (A, B) and counted by phase contrast light microscope under high power field (magnification, 100×), (C). All experiments were conducted in duplicates and similar results were repeated four times. The results are expressed as percentage of control and represent mean ± standard errors (SE) of the eight experiments. *p<0.05 significantly differs from PDGF-BB-stimulated cells (the fourth bar).

To determine the inhibitory effects of resveratrol on ARPE19 cell migration, we first performed ECIS migration assays. The results show that ARPE19 cell migration was promoted by PDGF-BB and the promotive effect was conspicuously suppressed by the preincubation of PDGF-BB with resveratrol. However, resveratrol did not inhibit ARPE19 cell migration in the samples without PDGF-BB (Figure 1). We also conducted Transwell migration assays to compare the results with the ECIS migration assays. Figure 2A shows that significant ARPE19 cell migration on fibronectin was observed in the absence of PDGF-BB, with about 20 migrated cells found in HPF, suggesting that fibronectin is a ‘chemoattractant’ for ARPE19 cells. PDGF-BB stimulated ARPE19 cell migration to about two folds over that elicited by fibronectin alone. However, resveratrol abolished the PDGF-BB-induced ARPE19 cell migration on fibronectin (Figure 2B). Quantitative analysis indicates that nearly 100% of migration was inhibited by 10 µM of resveratrol (Figure 2C). These observations indicate that resveratrol is effective in the prevention of PDGF-BB-induced ARPE19 cell migration.

### Resveratrol showed no cytotoxicity on ARPE19 cells

**Figure 3:**
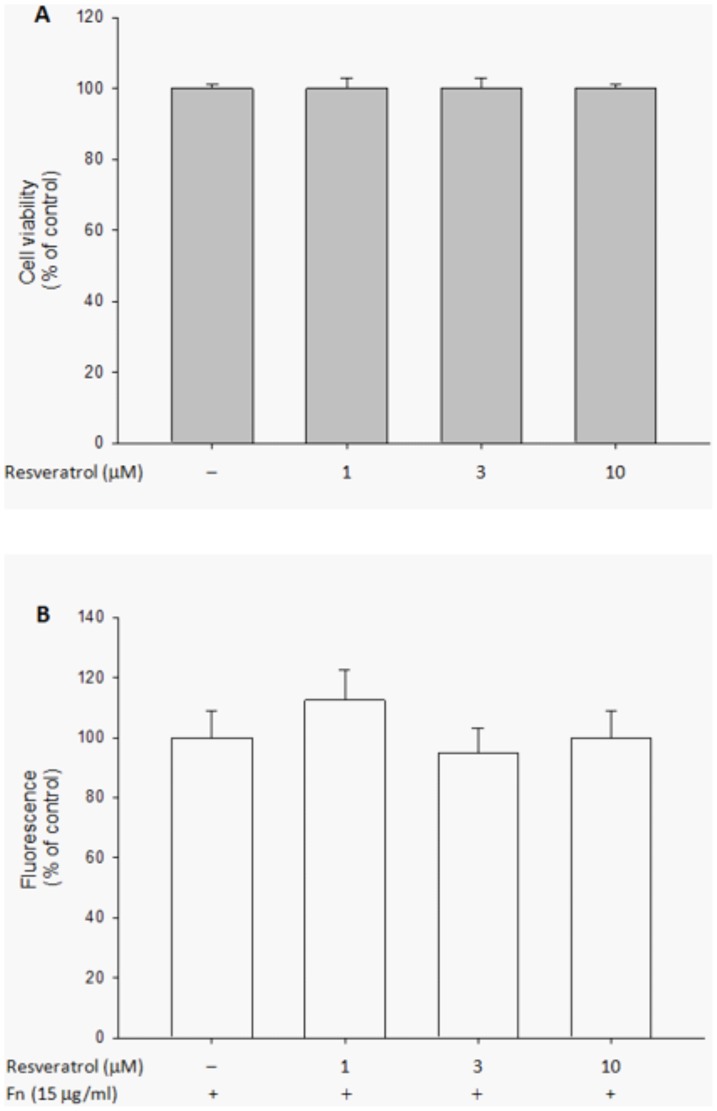
Viability and cell adhesion of ARPE19 cells was not influenced by resveratrol. The cells were treated with different concentrations of resveratrol for 24 hours after being starved for 24 hours. Cell viability was determined by MTT assay (A). BCECF-labeled cells were treated with DMSO or resveratrol for 30 minutes. They were then seeded and allowed to adhere on plates with precoated fibronectin (fn) (15 µg/ml) at 37°C for 1 hour. Fluorescence was measured using excitation and emission wavelength of 485 and 535 nm, respectively (B). The results are expressed as percentage of control and represent the mean ± standard errors (SE) of four independent experiments.

To exclude the possibility that resveratrol affects ARPE19 cell migration through its effects on cell viability, cell viability assays were performed. As shown in Figure 3A, resveratrol treatments (1, 3 and 10 µM) did not affect cell viability in MTT assays. The results indicate that resveratrol is safe for ARPE19 cells and its effects on ARPE19 cell migration did not result from the decrease of cell viability.

### Resveratrol showed no effect on ARPE19 cell adhesion

To determine whether resveratrol inhibited ARPE19 cell migration through interfering with their attachment to fibronectin, we tested the effect of resveratrol on ARPE19 cell adhesion with fibronectin coated. As shown in Figure 3B, the amount of cell adhesion was not affected by the presence of resveratrol. The observation indicates that the inhibitory effect of resveratrol on ARPE19 cell migration was not induced by interfering with the attachment of the cells to fibronectin.

### Resveratrol did not directly bind to platelet-derived growth factor-BB in dot binding assay

**Figure 4:**
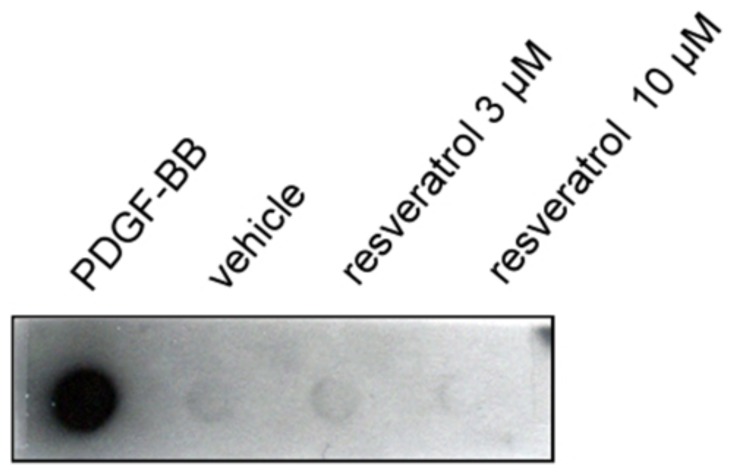
Resveratrol did not directly interact with PDGF-BB in dot binding assay. Human recombinant PDGF-BB, phosphate buffer saline (PBS) and the indicated concentrations of resveratrol were applied onto the nitrocellulose (NC) membrane. The membrane was incubated with PDGF-BB in PBS and then developed by probing with Ab directed against PDGF-BB. The results presented are representative of four independent experiments.

Recombinant human PDGF-BB and resveratrol were immobilized on the nitrocellulose (NC) membrane. After incubation with or without PDGF-BB, the membrane was further incubated with antibodies against PDGF-BB and then developed. We observed that immobilized PDGF-BB can be recognized by the anti-PDGF-BB Ab, suggesting the specificity of Ab. But the 3 µM and 10 µM of resveratrol did not directly bind to PDGF-BB. The data indicates that resveratrol cannot directly bind to PDGF-BB (Figure 4).

### Resveratrol inhibited PDGF-BB-induced PDGFRβ phosphorylation and downstream PI3K/Akt and MAPK pathway activation

**Figure 5:**
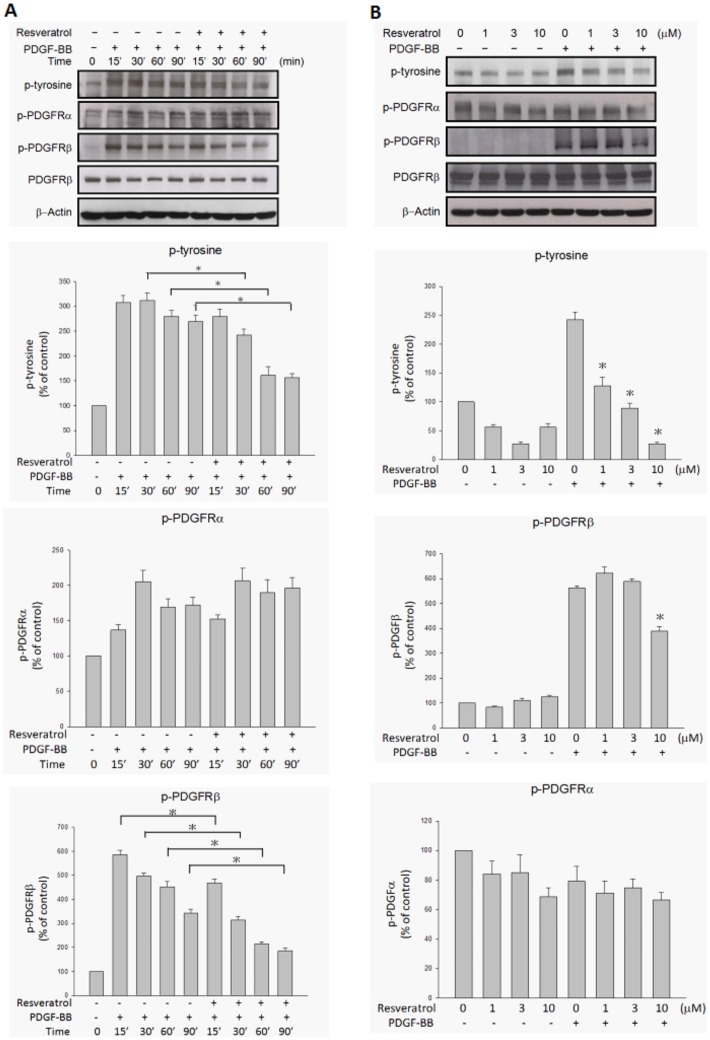
PDGF-BB-induced tyrosine and PDGFRβ phosphorylations were inhibited by resveratrol in a time- and concentration-dependant manner. ARPE19 cells were treated with the indicated lengths of time of PDGF-BB (20 ng/ml) and preincubated with or without resveratrol (10 µM) at 37°C (A). After being further preincubated for the indicated concentrations of resveratrol and incubated with or without PDGF-BB (20 ng/ml) at 37°C for 30 minutes, the cells were collected and their lysates were analyzed by Western blot analysis (B). The changes in phosphorylated tyrosine, PDGFRα and PDGFRβ expression were evaluated. The quantitative data of western blot are shown below the panels which are expressed as percentage of control and represent mean ± standard errors (SE) of the four independent experiments. *p<0.05 significantly differs from same indicated time of cells stimulated PDGF-BB only (A) and *p<0.05 significantly differs from PDGF-BB-stimulated cells (the fifth bar) (B).

**Figure 6:**
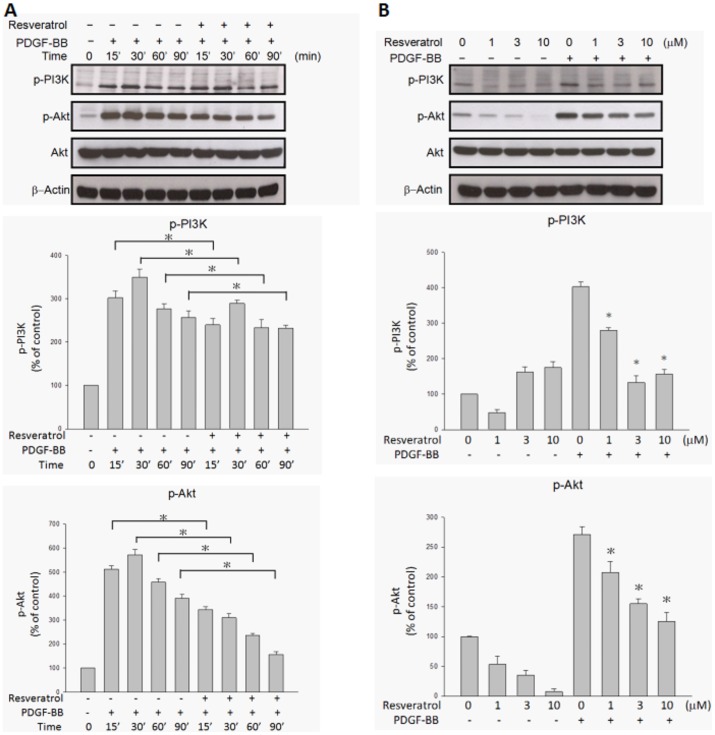
PDGF-BB-induced PI3K and Akt phosphorylations were inhibited by resveratrol in a time- and concentration-dependant manner. ARPE19 cells were treated with the indicated lengths of time of PDGF-BB (20 ng/ml) and preincubated with or without resveratrol (10 µM) at 37°C (A). After being further preincubated for the indicated concentrations of resveratrol and incubated with or without PDGF-BB (20 ng/ml) at 37°C for 30 minutes, the cells were collected and their lysates were analyzed by Western blot analysis (B). The changes in phosphorylated PI3K and Akt expression were evaluated. The quantitative data of western blot are shown below the panels which are expressed as percentage of control and represent mean ± standard errors (SE) of the four independent experiments. *p<0.05 significantly differs from same indicated time of cells stimulated PDGF-BB only (A) and *p<0.05 significantly differs from PDGF-BB-stimulated cells (the fifth bar) (B).

**Figure 7:**
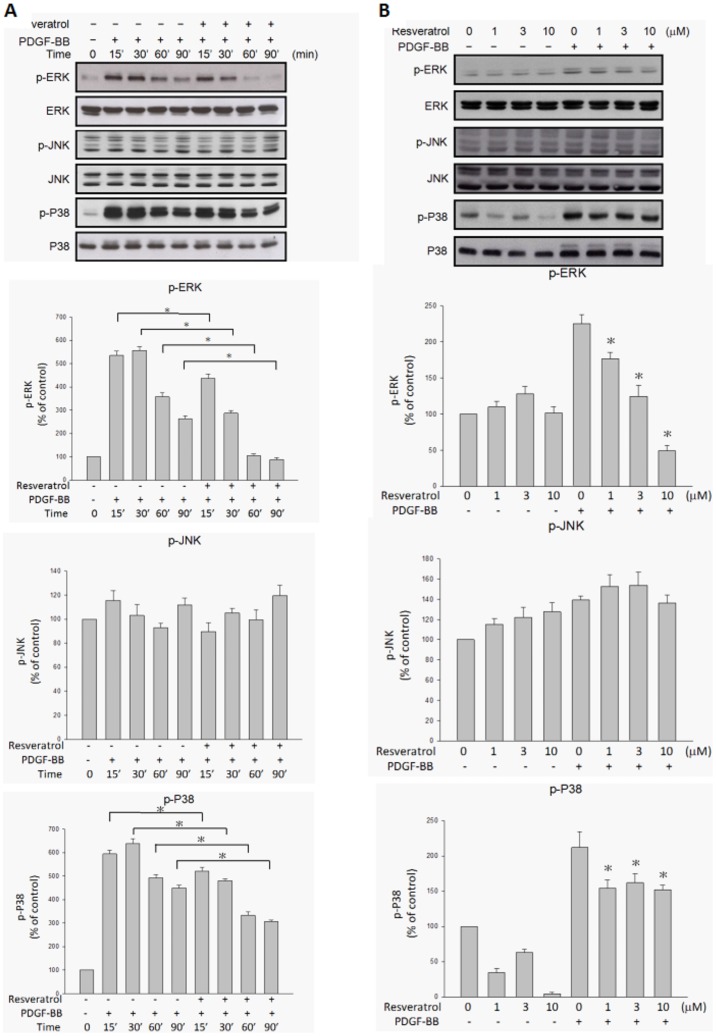
PDGF-BB-induced ERK and P38 phosphorylations were inhibited by resveratrol in a time- and concentration-dependant manner. ARPE19 cells were treated with the indicated lengths of time of PDGF-BB (20 ng/ml) and preincubated with or without resveratrol (10 µM) at 37°C (A). After being further preincubated for the indicated concentrations of resveratrol and incubated with or without PDGF-BB (20 ng/ml) at 37°C for 30 minutes, the cells were collected and their lysates were analyzed by Western blot analysis (B). The changes in phosphorylated ERK, JNK and p38 expression were evaluated. The quantitative data of western blot are shown below the panels which are expressed as percentage of control and represent mean ± standard errors (SE) of the four independent experiments. *p<0.05 significantly differs from same indicated time of cells stimulated PDGF-BB only (A) and *p<0.05 significantly differs from PDGF-BB-stimulated cells (the fifth bar) (B).

It has been reported that PDGF-BB binding to PDGF receptors (PDGFR) is associated with the dimerization, autophosphorylation, and activation of PDGFR-tyrosine kinase activity[28]. To determine whether PDGF-BB-induced signaling pathways are affected by resveratrol, the extent of phosphorylation of PDGFR and its downstream components in ARPE19 cells was evaluated. Figure 5 shows that stimulation of ARPE19 cells with PDGF-BB resulted in PDGFRβ phosphorylation, as determined by Western blotting with antibody directed against tyrosine, PDGFRβ. Preincubation of PDGF-BB with resveratrol resulted in the inhibition of tyrosine, PDGFRβ phosphorylation in a time- and concentration-dependent manner. There was no PDGFRα phosphorylation under PDGF-BB stimulation or resveratrol preincubation. Figure 6 shows that PI3K and Akt phosphorylation were also increased by PDGF-BB stimulation. Preincubation of PDGF-BB with resveratrol resulted in the decrease of PI3K and Akt phosphorylation in a time- and concentration-dependent manner. Among MAPKs, ERK and p38 phosphorylation increased after being stimulated with PDGF-BB and the increase was suppressed by preincubation of PDGF-BB with resveratrol in a concentration-dependent manner (Figure 7), while JNK phosphorylation was not influenced by PDGF-BB stimulation or resveratrol preincubation.

### PDGF-BB-induced cell migration was inhibited by resveratrol and by suppression of PDGFR, PI3K/Akt and MAPK signaling

**Figure 8:**
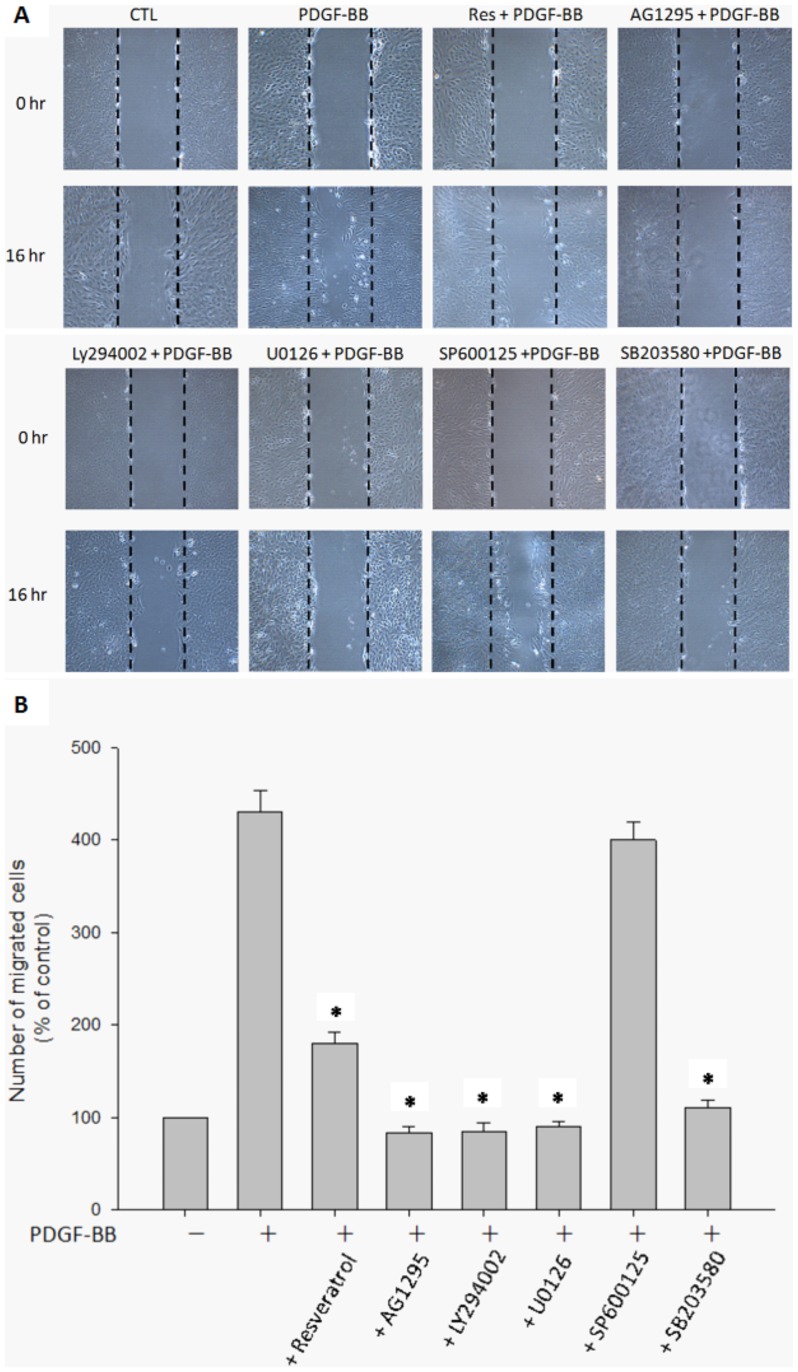
PDGF-BB-induced cell migrations were inhibited by resveratrol and by suppression of PDGFR, PI3K/Akt and MAPK signaling. The plates with confluent monolayer of ARPE cells were pretreated with mitomycin-C (5 µg/ml) for 1 hour, then wounded with a linear scratching by a sterile 20- µl pipette tip. The cells were immediately washed and were incubated with PDGF-BB (20 ng/ml) only, PDGF-BB in combination with resveratrol (10 µM), AG1295 (10 µM), LY294002 (10 µM), U0126 (10 µM), SP600125 (3 µM) and SB203580 (3 µM) respectively. The wound closure was monitored for 16 h followed by photography under phase-contrast microscope (x100) (A). The quantitative data of the number of migrated cell in the wound area are expressed as percentage of control and represent mean ± standard errors (SE) of the four independent experiments. *p<0.05 significantly differs from PDGF-BB-stimulated cells (the second bar) (B).

The migration of ARPE19 cells with various treatments was assessed using a scratch wound healing assay. Incubation of ARPE19 with PDGF-BB brought about an increase in the migration of ARPE19 cells. Then, we assessed the effects of resveratrol, PDGFR tyrosine kinase blocker inhibitor AG1295, PI3K inhibitor LY294002, MEK inhibitor U0126, JNK inhibitor SP600125 and P38 inhibitor SB203580 on PDGF-BB-induced migration of RPE cells. Figure 8 shows enhanced migration of RPE cells with significant wound closure by 16 h in the plates incubated with PDGF-BB (20 ng/ml) only, but a significant area of the wound remaining uncovered in the plates treated with resveratrol (10 µM) in the presence of PDGF-BB. Moreover, the suppression of PDGF-BB induced cell migration was observed in the plates incubated with AG1295 (10 µM), LY294002 (10 µM), U0126 (10 µM) and SB203580 (3 µM) respectively, indicating the possible involvement of the PI3K/Akt, ERK and p38 pathways and the signaling events by which resveratrol blocks PDGF-BB-induced RPE cell migration. Incubation with SP600125 (3 µM) did not show the inhibitory effect in RPE migration in the presence of PDGF-BB. Our result shows that the PDGF-BB-induced migration of ARPE19 cells is inhibited by resveratrol and mediated through PDGFR, PI3K/Akt, ERK and p38 signaling.

## Discussion

There are two PDGF peptides encoded by two different genes: PDGF-A and PDGF-B. The mature peptides are approximately 100 aa long and share 60% sequence similarity. The biologically active PDGF molecules are either homodimers (PDGF-AA and PDGF-BB) or heterodimers (PDGF-AB) that are formed by two disulphide bonds between the monomers[29], [30], [31]. PDGF dimers exert their effects on target cells by binding to cell surface receptors (PDGFRα and PDGFRβ) that contain five IgG-like domains extracellularly and an intracellular tyrosine kinase domain. PDGFRα binds to both A and B chains while PDGFRβbinds only to B chain with high affinity. PDGFR dimerization is driven by ligand binding; PDGF-AA induces the αα receptor dimers, AB the αα and αβ receptor dimers, and BB all three combinations of receptor dimers[32]. The expression of PDGF-A and -B genes and their receptors are independently regulated, and depend on cell types and physio-pathological conditions[30]. Recently, two new PDGF genes, PDGF-C and PDGF-D have been discovered, and their protein products are secreted as latent factors that require activation by proteolysis[33]. PDGF-C and -D form only homodimers that bind to PDGFRα and PDGFRβ, respectively, with high affinity. Although the expression of PDGF-C and -D has been demonstrated in a variety of cells, including cancer cells, their functional significance and physiological roles are much less understood than PDGF-A and -B.

Proliferative vitreoretinal diseases, such as PVR and PDR, are major causes of retinal detachment and result from the formation of fibrotic epiretinal membranes either on the surface of the retina or within the vitreous[34], [35]. Elevated expression of PDGF has been observed in RPE cells after retinal detachments or retinal laser treatment in murine model systems[36]. Expression of PDGF-A, -B and PDGFRβ has also been shown in *in vitro* wounded human RPE cell cultures[36]. In immunohistochemical studies with epiretinal membranes isolated from PVR and PDR patients, PDGF and PDGFRαwere found to be elevated[7], [37]. The role of PDGF in PVR has been clearly demonstrated in transgenic mice expressing PDGF-A or PDGF-B in photoreceptors by using rhodopsin promoter[38], [39]. PDGF-A overexpression led to proliferation of glial cells and traction retinal detachment without the involvement of vascular cells. PDGF-B overexpression resulted in traction retinal detachment involving proliferation of both vascular and non-vascular cells similar to that observed in diabetic retinopathy[39] In this PDGF transgenic mouse model, the kinase inhibitor PKC412 was shown to suppress ERM formation and retinal detachment[40]. In studies involving experimental PVR in rabbits, PDGF receptor kinase inhibitor has been shown to attenuate PVR significantly[41]. Moreover, dominant negative mutants of PDGFRαcan attenuate the development of PVR[42], [43] and blocking signaling events by which the non-PDGFs indirectly activated PDGFRα protected rabbits from developing PVR[44]. It has been shown that even though RPE cells express PDGFRα substantially less than fibroblasts, they significantly boost PVR-related signaling events, cellular responses, and the PVR potential of ARPE19 cells[44], [45]. In FVM membrane of PDR, the contribution of RPE cells has been confirmed by ultrastructural investigation[46], [47]. The RPE layer is known to undergo the earliest pathological change in the diabetic retina[48]. Furthermore, patients with PDR typically have 5%–20% of RPE cells in combined traction rhegmatogenous retinal detachment membrane[46]. These observations indicate that RPE cells migrate through the retinal breaks to access the PDR membrane and may contribute to PDR progression by secreting angiogenic factors. High retinal expression of PDGF-B is observed homozygous rho/PDGF-B (rho/PDGF-BB) mice that results in traction retinal detachment from proliferation of both vascular and nonvascular cells, similar to diabetic retinopathy in humans[39]. It has been shown that a single intravitreous injection of an aptamer that specifically binds to PDGF-B is able to significantly reduce epiretinal membrane formation and retinal detachment in rho/PDGF-B mice[49]. Elevated concentration of PDGF-BB in vitreous fluid is seen in diabetic retinopathy and central retinal vein occlusion[50]. The level of PDGF isoforms in the vitreous, but not in serum, has been shown to correlate to the pathology of PDR. Thus, down-regulation of PDGF isoforms offers a potential target for the treatment of PDR[51].

Resveratrol, a stilbenoid compound found in red grapes and red wine, has shown inhibitory effects on cell migration in different cell lines. It can effectively and efficiently suppress endothelial cell proliferation and migration, with low cytotoxicity, in the ARPE19 and HUVEC lines[52]. Moreover, there have been observations of inhibitory effects on smooth muscle cell migration[53] and tumor necrosis factor-alpha-induced monocyte adhesion and migration[54]. Furthermore, resveratrol has been shown to inhibit endothelial cell migration and monocyte monocyte chemoattractant protein-induced (MCP-1) monocytic cell chemotaxis[55]. In breast-cancer cells, resveratrol has been reported to inhibit migration and invasion of cells through the suppression of the activation of PI3K/Akt signaling pathway[56], and epidermal growth factor (EGF)-induced migration, presumably through the EGFR/PI3K signaling pathway[57]. Through inhibition of PDGF signaling, vitisin B, the resveratrol tetramer, has been shown to inhibit cell migration in cultured vascular smooth muscle cells[58].

In the early stages of PVR, provisional ECM components including fibronectin are synthesized and deposited on the retinal surfaces[59]. Movement of an individual cell from a resting ECM substrate to a provisional ECM requires initial cell attachment to the new matrix, followed by cell spreading, stable attachment, and then migration. After RPE cells establish adhesion, migration through a retinal hole into the vitreous is a critical stage of PVR formation. PDGF has been known as a strong chemotactic factor for RPE cells in the presence of fibronectin[60], [61]. In the present study we found that resveratrol significantly inhibit PDGF-BB-induced RPE cell migration without any signs of cytotoxicity. Chemotactic migration is a complex phenomenon involving adhesion to ECM, cell motility, and the effects of chemotactic factors[62], [63], [64]. However, our results indicate that resveratrol does not affect RPE cell adhesion to fibronectin. The inhibition of PDGF-BB-induced cell migration by resveratrol and the specific inhibitors PDGFR, PI3K, MEK or p38 in scratch wound healing assay coincided with reduced activation of PDGF-BB-induced PDGFRβ, PI3K/Akt, ERK and p38 phosphorylation in Western blot analysis, suggesting that resveratrol inhibits cell migration via inhibition of the PDGFRβ, PI3K/Akt and MAPKs cascade.

In our system, preincubation of PDGF-BB with resveratrol resulted in a marked inhibition of its signaling in ARPE19 cells, including phosphorylation of PDGFRβ. Our previous studies have revealed the inhibitory effects of lycopene and EGCG on PDGF-BB-induced signaling and migration in ARPE19 cells[23], [24]. Our present findings suggest resveratrol influences PDGF-BB's function through similar mechanisms of action in ARPE19 cells. It directly binds to PDGF-BB, resulting in the blocking of PDGF-BB's interaction with its receptors, and inactivates PDGF-BB functions.

As described earlier, proliferation and migration of RPE cells are proposed to play a significant role in PVR and FVM progression in PDR. Though resveratrol has been shown to have the capability to inhibit migration of cancer cells, whether it possesses the same capability for normal cells has not been established. In this study, resveratrol is shown to inhibit PDGF-BB-induced migration and signaling in ARPE19 cells. One possible mechanism of action is the direct interaction with PDGF-BB. Further analysis showed that resveratrol inhibited PDGF-BB-induced signaling in ARPE19 cells. The findings and concepts presented here provide an important basis for further investigations to better understand the action mechanisms of resveratrol in RPE cells and possibly its beneficial effect on the prevention of FVM in PDR and PVR.
